# Computational Modelling of Cerebral Blood Flow Rate at Different Stages of Moyamoya Disease in Adults and Children

**DOI:** 10.3390/bioengineering10010077

**Published:** 2023-01-06

**Authors:** Surhan Bozkurt, Selim Bozkurt

**Affiliations:** 1 Department of Electronics and Automation, Istanbul Aydin University, Istanbul 34295, Turkey; 2 School of Engineering, Ulster University, Belfast BT15 1AP, UK

**Keywords:** Moyamoya disease, cerebral circulation, coarctation of the aorta, paediatrics

## Abstract

Moyamoya disease is a cerebrovascular disorder which causes a decrease in the cerebral blood flow rate. In this study, a lumped parameter model describing the pressures and flow rates in the heart chambers, circulatory system, and cerebral circulation with the main arteries in the circle of Willis, pial circulation, cerebral capillaries, and veins was used to simulate Moyamoya disease with and without coarctation of the aorta in adults and children. Cerebral blood flow rates were 724 mL/min and 1072 mL/min in the healthy adult and child cardiovascular system models. The cerebral blood flow rates in the adult and child cardiovascular system models simulating Moyamoya disease were 676 mL/min and 1007 mL/min in stage 1, 627 mL/min and 892 mL/min in stage 2, 571 mL/min and 831 in stage 3, and 444 and 537 mL/min in stage 4. The cerebral blood flow rates were 926 mL/min and 1421 mL/min in the adult and child cardiovascular system models simulating coarctation of the aorta. Furthermore, the cerebral blood flow rates in the adult and child cardiovascular system model simulating Moyamoya disease with coarctation of the aorta were 867 mL/min and 1341 mL/min in stage 1, 806 mL/min and 1197 mL/min in stage 2, 735 mL/min and 1121 in stage 3, and 576 and 741 mL/min in stage 4. The numerical model utilised in this study can simulate the advancing stages of Moyamoya disease and evaluate the associated risks with Moyamoya disease.

## 1. Introduction

Moyamoya disease is a disorder affecting cerebral arteries and causes a decrease in the cerebral blood flow rate [1]. It progresses slowly and can be divided into four stages. The focal intracranial stenosis of internal carotid arteries occurs in Stage 1 [2]. In stage 2, internal carotid arteries may be occluded along with anterior and middle cerebral arteries [2]. In stage 3, stenosis becomes more severe in the anterior and middle cerebral arteries [2]. In stage 4, stenosis occurs in the posterior cerebral arteries [3].

Moyamoya disease primarily affects children under 10; however, it may also occur in adults, mainly over 40 years old [4]. The general symptoms of Moyamoya disease are intracranial bleeding in adults and weakness of the limbs in children [5]. Furthermore, more than 6% of strokes in children occur because of Moyamoya disease [6]. The clinical outcome of Moyamoya disease may be different in adults and children. It results in ischemic strokes, transient ischemic attacks, intracerebral haemorrhages, seizures, and headaches [7]. Different factors such as inflammation, angiogenesis, vasculogenesis, and genetic factors may cause Moyamoya disease [8]. Moyamoya disease is also associated with other diseases, such as coarctation of the aorta [9,10,11,12], whilst coarctation of the aorta causing Moyamoya disease may be associated with mitral or aortic stenosis or congenital heart disease [13]. Therefore, different treatment methods have been used in patients with Moyamoya disease [14,15].

Mathematical modelling has been used to describe blood flow rates in different physiological cases and may also help to understand the effect of altered blood flow in patients with Moyamoya disease. Mathematical modelling of Moyamoya disease generally has been performed to study local blood flow effects in adult cerebral circulation. For instance, a computer model was developed to simulate the blood flow rate in the middle cerebral artery in Moyamoya disease and evaluate potential treatment techniques [16]. Computational fluid dynamics were utilised to simulate the effects of the bypass on internal carotid arteries in Moyamoya disease [17]. Computational fluid dynamics analyses were also utilised to simulate cerebral ischemia in patients with early stages of Moyamoya disease [18]. Blood flow due to bilateral intimal thickening of the distal internal carotid arteries in cerebral circulation was simulated using a 2D computational model to evaluate the effect of Moyamoya disease [19]. A mathematical model describing the blood flow rates in the main cerebral arteries will help to evaluate haemodynamic outcomes and risk factors for every stage of Moyamoya disease and understand the distribution of blood flow rate in cerebral arteries in adults and children.

In this study, a lumped parameter model simulating cardiac function, systemic and pulmonary circulations, and cerebral circulation was utilised to evaluate cerebral blood flow rate distribution in the advancing stages of Moyamoya disease with and without coarctation of the aorta for adults and children. The proposed model includes systemic and pulmonary circulatory systems and a complete configuration of the circle of Willis. Modelling the overall circle of Willis and the main cerebral arteries allows simulating the effect of the change of blood flow rate in one compartment on the other compartments.

## 2. Materials and Methods

The numerical model simulates cardiac function and systemic, pulmonary, and cerebral circulations. The left-ventricular pressure (*p_lv_*) includes active and passive components (*p_lv,a_, p_lv,p_*).

*p_lv_* = *p_lv,a_* + *p_lv,p_*. (1)

The left-ventricular contraction was driven by a function (*f_act,lv_*) whilst end-systolic elastance (*E_es,lv_*) and left-ventricular volume (*V_lv_*) were used to obtain the left-ventricular active pressure (*p_lv,a_).*

(2) $$p_{lv,a}\left(t\right)=E_{es,lv}\left(V_{lv}-V_{lv,0}\right)f_{act,lv\;}\left(t\right).$$

The left-ventricular passive pressure (*p_lv,p_*) was simulated using an exponential function which utilises the left-ventricular volume (*V_lv_*) and additional parameters (*A_lv_*, *B_lv_*).

(3) $$p_{lv,p}=A_{lv}\left[e^{B_{lv}\left(V_{lv}-V_{lv,0}\right)}-1\right].$$

The left-ventricular volume (*V_lv_*) was a function of the left-ventricular radius (*r_lv_*), the left-ventricular long axis length (*l_lv_*), and a scaling parameter (*K_lv_*).

(4) $$V_{lv}=\frac{\left(4/3\right)\pi K_{lv}r_{lv}^{2}l_{lv}}{2}.$$

The derivative of the left-ventricular radius over time (d*r_lv_*/*dt*) was modelled with the left-ventricular volume (*V_lv_*), left-ventricular long axis length (*l_lv_*), the scaling coefficient (*K_lv_*), and the flow rates through the mitral and aortic valves (*Q_mv_*, *Q_av_*).

(5) $$\frac{dr_{lv}}{dt}=\frac{3\left(Q_{mv}-Q_{av}\right)}{4\pi K_{lv}l_{lv}}{\left(\frac{6V_{lv}}{4\pi K_{lv}l_{lv}}\right)}^{-1/2}.$$

The left-atrial pressure was modelled using the left-atrial elastance function (*E_la_*(*t*)), left-atrial volume, and zero-pressure volume (*V_la_*, *V_la,0_*).

(6) $$p_{la}\left(t\right)=E_{la}\left(t\right)\left(V_{la}-V_{la,0}\right).$$

The left-atrial volume (*V_la_*) was a function of the left-atrial radius (*r_la_*), the long axis length (*l_la_*), and a scaling parameter (*K_la_*). The derivative of the left-atrial radius over time (d*r_la_*/dt) was simulated by the pulmonary venous and mitral valve flow rates (*Q_vp_, Q_mv_*), the left-atrial volume (*V_la_*), the long axis length (*l_la_*), and the coefficient *K_la_*.

(7) $$V_{la}=\frac{2}{3}\pi K_{la}r_{la}^{2}l_{la}.$$

(8) $$\frac{dr_{la}}{dt}=\frac{3(Q_{vp}-Q_{mv})}{4\pi K_{la}l_{la}}{\left(\frac{3V_{la}}{2\pi K_{la}l_{la}}\right)}^{-1/2}.$$

The right-atrium and right-ventricle functions were simulated similarly, whereas the parameter values in these compartments were different. Heart valves allowed only one-way blood flow; blood flow rates through them were described by the pressure across the valve and valve resistances (*R*). The mitral valve flow rate (*Q_mv_*) is provided below.

(9) $$Q_{mv}=\frac{p_{la}-p_{lv}}{R_{mv}}.$$

Blood circulation was described using a 0D model, which included electrical analogues for resistance (*R*), compliance (*C*), and inertia (*L*) in the blood vessels. Changes in the aortic blood pressure and flow rate over time (d*p_ao_*/dt, *Q_ao_*/dt) are given below.

(10) $$\frac{dp_{ao}}{dt}=\frac{Q_{av}-Q_{ao}}{C_{ao}},$$

(11) $$\frac{dQ_{ao}}{dt}=\frac{p_{ao}-p_{as}-R_{ao}Q_{ao}}{L_{ao}},$$

where *Q_av_* and *p_as_* are the aortic valve flow rate and systemic arteriolar pressure. *C_ao_*, *R_ao_*, and *L_ao_* represent aortic compliance, resistance, and inertance.

**Figure 1:**
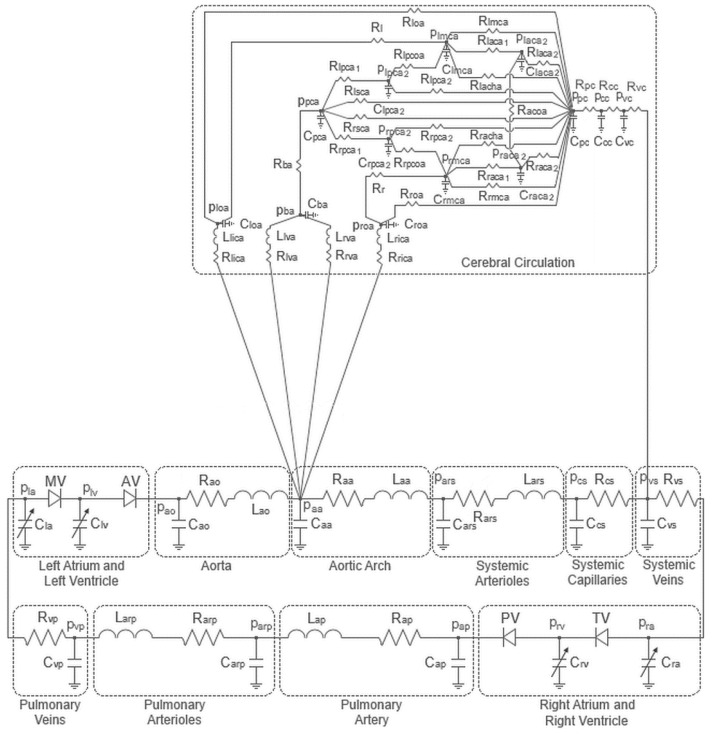
Circuit diagram representation of the cardiovascular system model.

The cerebral circulation includes the cerebral arteries and circle of Willis, pial arterioles, cerebral capillaries, and cerebral veins. The circuit diagram representation of the cardiovascular system is given in Figure 1. Values of the parameters used in the adult and child cardiovascular system models are given in Supplementary Table S1. Detailed information about cardiac function and cerebral circulation modelling can be found in [20,21,22].

Flow rates, pressures, and heart chamber volumes are the unknowns being solved in the current model, whereas total blood volume is the input of the system. Resistances, inertances, and compliances in the blood vessels and heart chamber elastances affect the parameters being solved in the model.

The aortic stiffness increases and the aortic elasticity decreases in patients with coarctation of the aorta [23]. Therefore, the systemic arteriolar resistance was increased, and the compliances of the aorta, aortic arch, and systemic arterioles were decreased in the cardiovascular system models simulating coarctation of the aorta. Furthermore, arterial elasticity is lower in children than in adults [24]. Lower compliance values were used in the child cardiovascular system model. The systemic and pulmonary circulatory system parameters used in the adult and child cardiovascular system models are given in Supplementary Table S2.

Stenosis occurs in the internal carotid arteries in patients with stage 1 Moyamoya disease. The severity of stenosis increases in the anterior and middle cerebral and internal carotid arteries at stage 2 Moyamoya disease. The severity of stenosis of the internal carotid, anterior cerebral, and middle cerebral arteries further increases at stage 3 Moyamoya disease. Furthermore, stenosis occurs in the posterior cerebral arteries and the other cerebral blood vessels at stage 4 [2,3]. The cerebral circulatory system model resistances were adjusted manually, considering blood flow rates in cerebral circulation at different stages of Moyamoya disease [2,3].

The parameter values used in the cerebral circulatory system for stages of Moyamoya disease are given in Supplementary Table S3 for the adult cardiovascular system model and Supplementary Table S4 for the child cardiovascular system model. Parameter values in Supplementary Table S3 were taken from the literature [21,22,25] to simulate blood flow in the adult cardiovascular system model. Parameter values in Supplementary Table S4 were adapted from the adult cardiovascular system model to simulate blood flow in the child cardiovascular system model considering the total cerebral blood flow rates given in [26].

Heart rates in the adult and child cardiovascular system models were adjusted as 75 bpm and 80 bpm, respectively [20]. The simulations were performed using Matlab Simulink 2017a. All equations were solved using the ode15s solver. The maximum step size was 1 × 10^−3^ s, and the relative tolerance was 1 × 10^−3^.

## 3. Results

**Figure 2:**
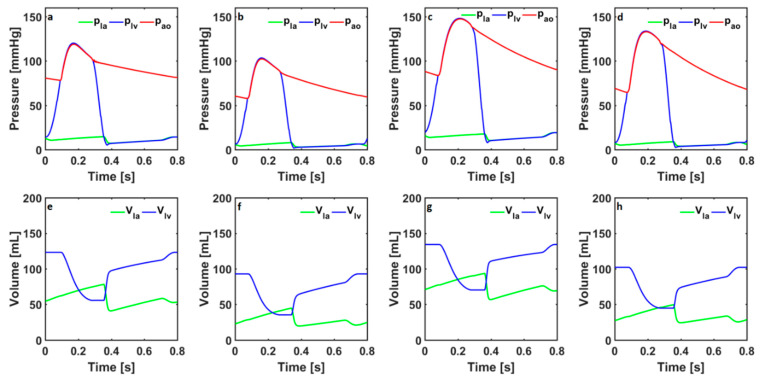
The left-atrial and left-ventricular pressures (p_la_, p_lv_) and aortic pressure (p_ao_) in the cardiovascular system models simulate healthy conditions in (**a**) adults and (**b**) children and coarctation of the aorta in (**c**) adults and (**d**) children. The left-atrial and left-ventricular volumes (V_la_, V_lv_) in the cardiovascular system models simulate healthy conditions in (**e**) adults and (**f**) children and coarctation of the aorta in (**g**) adults and (**h**) children.

Blood pressures in the left atrium, left ventricle, and aorta, as well as the left-atrial and left-ventricular volumes, in the cardiovascular system models simulating a healthy condition and coarctation of the aorta in adults and children are given in Figure 2.

The systolic left-ventricular pressures were 120 mmHg and 103 mmHg in the numerical models simulating healthy conditions in adults and children. The aortic pressure changed between 78 mmHg and 119 mmHg in the numerical model simulating healthy conditions in adults. The aortic pressure changed between 58 mmHg and 102 mmHg in the numerical model simulating healthy conditions in children. The systolic left-ventricular pressures in the adult and child cardiovascular system models simulating coarctation in the aorta were 148 mmHg and 134 mmHg, respectively. In the adult cardiovascular system model, the aortic pressure changed between 84 mmHg and 147 mmHg. The aortic pressure changed between 64 mmHg and 133 mmHg in the child cardiovascular system model.

The left-ventricular volume in the healthy adult cardiovascular system model changed between 56 mL and 123 mL, whereas, in the healthy child cardiovascular system model, it changed between 35 mL and 93 mL. The left-ventricular volume in the adult cardiovascular system model simulating coarctation of the aorta changed between 70 mL and 135 mL. The left-ventricular volume in the child cardiovascular system model simulating coarctation of the aorta changed between 45 mL and 102 mL.

**Figure 3:**
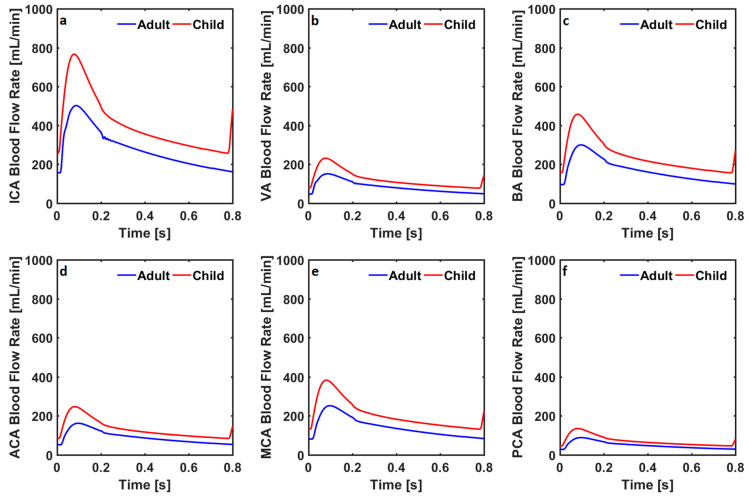
Blood flow rates through (**a**) the internal carotid arteries (ICA), (**b**) the vertebral arteries (VA), (**c**) the basilar artery (BA), (**d**) the anterior cerebral arteries (ACA), (**e**) the middle cerebral arteries (MCA), and (**f**) the posterior cerebral arteries (PCA) in the numerical model simulating healthy conditions in adults and children.

Blood flow rates in the internal carotid arteries, vertebral arteries, basilar artery, and anterior, middle, and posterior cerebral arteries in the adult and child cardiovascular system models simulating a healthy condition are given in Figure 3.

**Figure 4:**
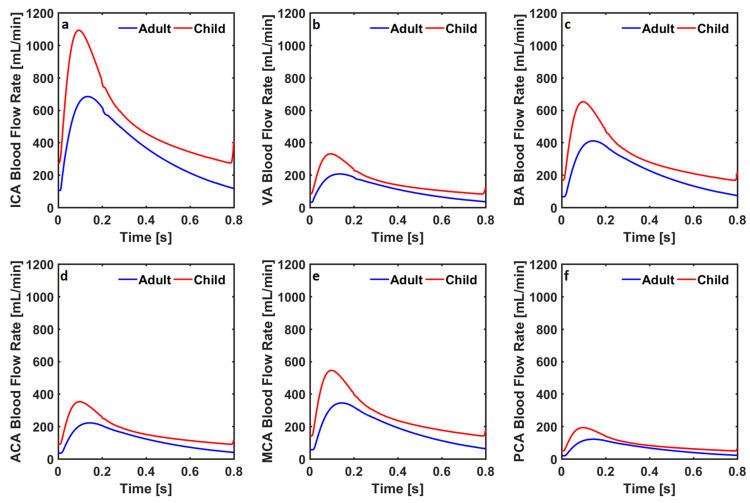
Blood flow rates through (**a**) the internal carotid arteries (ICA), (**b**) the vertebral arteries (VA), (**c**) the basilar artery (BA), (**d**) the anterior cerebral arteries (ACA), (**e**) the middle cerebral arteries (MCA), and (**f**) the posterior cerebral arteries (PCA) in the cardiovascular system models simulating coarctation of the aorta in adults and children.

The blood flow rate through the internal carotid arteries changed between 156 mL/min and 510 mL/min in the healthy adult cardiovascular system model, whereas it changed between 258 mL/min and 766 mL/min in the healthy child cardiovascular system model. The blood flow rate through the vertebral arteries changed between 47 mL/min and 151 mL/min, and between 78 mL/min and 232 mL/min, respectively, in the healthy adult and child cardiovascular system models. The blood flow rate through the basilar artery changed between 96 mL/min and 299 mL/min, and between 156 mL/min and 457 mL/min in the healthy adult and child cardiovascular system models. Blood flow rates through the anterior, middle, and posterior cerebral arteries in the healthy adult cardiovascular system model changed between 52 mL/min and 162 mL/min, between 81 mL/min and 251 mL/min, and between 28 mL/min and 88 mL/min. Blood flow rates through anterior, middle, and posterior cerebral arteries in the healthy child cardiovascular system model changed between 84 mL/min and 247 mL/min, between 132 mL/min and 383 mL/min, and between 46 mL/min and 135 mL/min. Blood flow rates in the internal carotid arteries, vertebral arteries, basilar artery, anterior cerebral arteries, middle cerebral arteries, and posterior cerebral arteries in the adult and child cardiovascular system models simulating coarctation of the aorta are given in Figure 4.

The blood flow rate through the internal carotid arteries changed between 105 mL/min and 681 mL/min in the cardiovascular system model simulating coarctation of the aorta for adults, whereas it changed between 274 mL/min and 1092 mL/min in the child cardiovascular system model simulating coarctation of the aorta. The blood flow rate through the vertebral arteries changed between 31 mL/min and 205 mL/min, and between 83 mL/min and 330 mL/min in the adult and child cardiovascular system models with coarctation of the aorta. The blood flow rate through the basilar artery changed between 66 mL/min and 408 mL/min, and between 167 mL/min and 652 mL/min in the adult and child cardiovascular system models simulating coarctation of the aorta.

**Figure 5:**
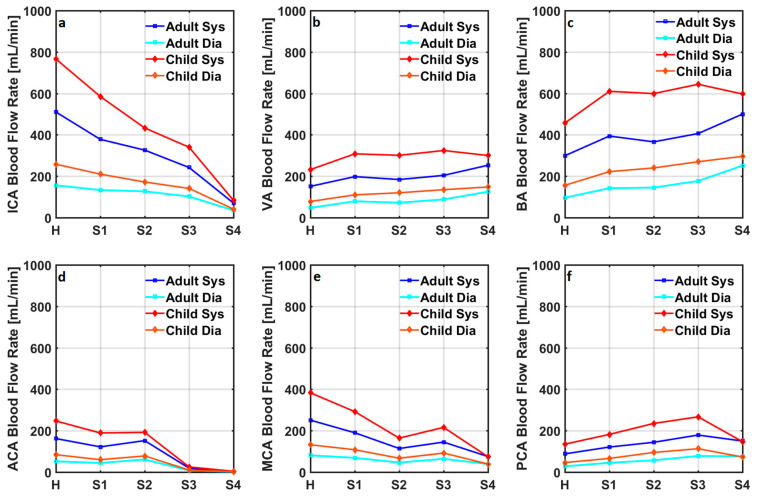
Systolic and diastolic cerebral blood flow rates in (**a**) internal carotid arteries (ICA), (**b**) vertebral arteries (VA), (**c**) basilar artery (BA), (**d**) anterior cerebral arteries (ACA), (**e**) middle cerebral arteries (MCA), and (**f**) posterior cerebral arteries (PCA) in the adult and child cardiovascular system models simulating a healthy condition and advancing stages of the Moyamoya disease. H, S, Sys, and Dia represent healthy, stage, systolic, and diastolic.

Blood flow rates through the anterior, middle, and posterior cerebral arteries in the healthy adult cardiovascular system model changed between 35 mL/min and 221 mL/min, between 56 mL/min and 343 mL/min, and between 19 mL/min and 121 mL/min. Blood flow rates through the anterior, middle, and posterior cerebral arteries in the healthy child cardiovascular system model changed between 90 mL/min and 352 mL/min, between 142 mL/min and 545 mL/min, and between 49 mL/min and 193 mL/min. Systolic and diastolic cerebral blood flow rates in internal carotid, vertebral, basilar, anterior cerebral, middle cerebral, and posterior cerebral arteries in the adult and child cardiovascular system models simulating a healthy physiological condition and advancing stages of Moyamoya disease are given in Figure 5.

Systolic and diastolic flow rates in the internal carotid arteries decreased with the progression of the Moyamoya disease in both adult and child cardiovascular system models. Vertebral arterial systolic and diastolic blood flow rates were relatively low in the cardiovascular system models simulating healthy conditions in adults and children. Although systolic and diastolic blood flow rates increased in the vertebral arteries, the progression of Moyamoya disease did not increase the blood flow rate at every stage. A similar change in the basilar arterial blood flow rates was also simulated. Anterior cerebral arterial systolic and diastolic blood flow rates were relatively high in the cardiovascular system models simulating healthy conditions in adults and children. The progression of Moyamoya disease did not remarkably affect the blood flow rates in the anterior cerebral arteries at stages 1 and 2.

**Figure 6:**
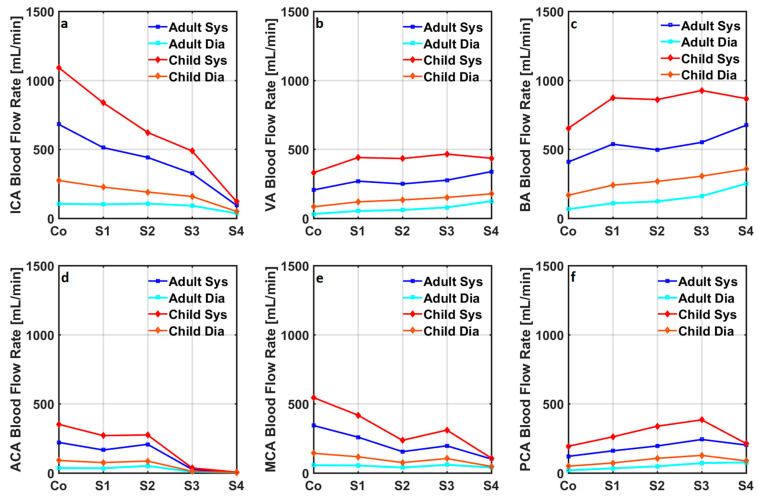
Systolic and diastolic cerebral blood flow rates in (**a**) internal carotid arteries (ICA), (**b**) vertebral arteries (VA), (**c**) basilar artery (BA), (**d**) anterior cerebral arteries (ACA), (**e**) middle cerebral arteries (MCA), and (**f**) posterior cerebral arteries (PCA) in the adult and child cardiovascular system models simulating a healthy condition and advancing stages of the Moyamoya disease together with coarctation of the aorta. H, S, Sys, and Dia represent healthy, stage, systolic, and diastolic.

In contrast, anterior cerebral arterial blood flow rates at the diastole and systole reduced significantly in stages 3 and 4 of Moyamoya disease in both adult and child cardiovascular system models. Systolic and diastolic middle cerebral arterial blood flow rates decreased in stage 1 and 2 Moyamoya disease, whilst they increased in stage 3 Moyamoya disease and decreased again in stage 4 Moyamoya disease. Progression of Moyamoya disease increased the systolic and diastolic posterior cerebral arterial blood flow rates until stage 3 in both adult and child cardiovascular system models. The progression of Moyamoya disease to stage 4 reduced the posterior cerebral arterial blood flow rates in both cardiovascular system models except the diastolic posterior cerebral arterial blood flow rate in the adult cardiovascular system model. Systolic and diastolic cerebral blood flow rates in the adult and child cardiovascular system models simulating advancing stages of Moyamoya disease with coarctation of the aorta are given in Figure 6.

Coarctation of the aorta resulted in increased systolic and diastolic blood flow rates in the cerebral circulation, whereas the progression of Moyamoya disease caused a similar change in the blood flow rates in each compartment as in the cardiovascular system models not simulating coarctation of the aorta. Systolic and diastolic aortic blood pressures, cardiac output, total cerebral blood flow rate, and mean blood flow rates through the internal carotid, vertebral, basilar, anterior cerebral, middle cerebral, and posterior cerebral arteries in the numerical models simulating healthy conditions and advancing stages of the Moyamoya disease with and without coarctation of the aorta are given in Table 1.

Progression of Moyamoya disease had little effect on the systolic and diastolic pressures in the aorta and cardiac output in both adult and child cardiovascular system models without coarctation of the aorta. On the other hand, the progression of Moyamoya disease had a more profound effect on the systolic and diastolic pressures in the aorta and cardiac output in the cardiovascular system models simulating coarctation of the aorta. Progression of Moyamoya disease decreased total cerebral blood flow rate in both adult and child cardiovascular system models with and without coarctation of the aorta, whereas total cerebral blood flow rate was relatively high in the cardiovascular system models simulating coarctation of the aorta.

## 4. Discussion

In this study, a lumped parameter model was utilised to simulate cerebral blood flow rates for advancing stages of Moyamoya disease with and without coarctation in the aorta for adults and children. The cerebral blood flow rate decreases with age [26,27]. The total cerebral blood flow rate in healthy adults varies within a wide range [28]. The average cerebral blood flow rate per 100 g of adults’ and children’s brains was reported as 50 mL/min and 92.1 mL/min, respectively [27]. It is assumed that the brain’s weight is 1400 g in adults and 1200 g in children aged between 8 and 12 [29]. Therefore, the average cerebral blood flow rate is around 700 mL/min in adults and 1105 mL/min in children aged between 8 and 12. The cardiovascular system models simulated the total cerebral blood flow rates of around 724 mL/min and 1072 mL/min for healthy adults and children, respectively. The cerebral blood flow rates in both adult and child decreases in the patients with Moyamoya disease [5,27]. The simulation results showed that the cerebral blood flow rate was consistent with reported clinical data in the cardiovascular system models simulating Moyamoya disease.

Blood vessels on the left and right sides of the circle of Willis were modelled using identical resistance and compliance values. Blood flow rates simulated in the numerical model were the same on the left and right sides of the cerebral circulation model. This configuration is valid for almost half of the population [30,31]. Therefore, this model provides a general outlook for a complete circle of Willis configuration. However, it can easily be modified to simulate the circle of Willis configuration with different properties or anatomical variations. It can provide information about how the flow rates in cerebral arteries are affected because of Moyamoya disease in different configurations of circle of Willis in adults and children.

Computational fluid dynamics (CFD) modelling in Moyamoya Disease can provide information about the blood velocities used to simulate patient-specific blood flow [18]. However, accurate results from CFD analyses require correct settings of boundary conditions at inlet and outlet sites of the computational domain. The developed model can provide flow rates and pressures at different stages of Moyamoya disease and can be used to simulate boundary conditions for CFD models. This is especially useful in children as the blood flow rates and pressures change associated with age [26,32]. Therefore, patient-specific boundary conditions are required to accurately simulate haemodynamics in this cohort. Such a task requires optimisation of the parameter values in the presented cardiovascular system and cerebral circulation model. Optimisation methods to estimate parameter patient-specific parameter values in adults and children have already been presented [33,34]. A similar framework can be utilised to optimise parameter values and simulate personalised blood flow rates in each modelled segment of cerebral circulation.

Transient ischemic attacks [35,36], speech difficulties [37], mental decline, loss of sensation, and cognitive impairments [38] are observed in children patients with Moyamoya disease. Cerebral ischemic attacks [35] and stroke [39] are observed in adult patients with Moyamoya disease. Although headache may occur in adults and children, it is observed more in children [38]. The blood flow rate through the anterior and middle cerebral arteries decreased by around 20% in adult and child cardiovascular system models due to the increase in the resistance of internal carotid arteries in stage 1. The decrease in blood flow rate through anterior cerebral arteries may cause weakness in the hands, limbs, and feet. Moreover, the decreased blood flow rate in the middle cerebral arteries may result in speech difficulties [37]. The resistance of the middle cerebral arteries was increased to simulate stage 2 Moyamoya disease. Increased middle cerebral arterial resistance also increased the blood flow rate in the anterior cerebral arteries, whereas the middle cerebral arterial flow rate decreased. Therefore, the risk of having speech difficulties may increase in Moyamoya disease at stage 2.

The resistance of anterior cerebral arteries was increased to simulate stages 3 and 4 of Moyamoya disease, along with the resistance of internal carotid arteries. The blood flow rates through the internal carotid, anterior, and middle cerebral arteries decreased remarkably in stages 3 and 4 of Moyamoya disease. The decrease in blood flow rate through anterior cerebral arteries may cause transient ischemic attacks in children and cerebral ischemic attacks in adults. Furthermore, it may cause stroke in adults. Additionally, a decrease in blood flow rates through middle cerebral arteries may cause cognitive impairments in adults and children.

Blockage of the internal carotid, anterior, and middle cerebral arteries may increase the blood flow rate through the vertebrobasilar system. The blood flow rates through vertebral and basilar arteries increased in adult and child cardiovascular system models simulating stages 1, 2, and 3 of Moyamoya disease. The increased blood flow rate through the vertebral and basilar arteries may cause an aneurysm and bleeding in the vertebrobasilar [40] and blindness [36,38] in Moyamoya disease due to increased pressure. The blood flow rates through vertebral and basilar arteries decreased in the adult and child cardiovascular system model, simulating stage 4 Moyamoya disease.

Posterior cerebral artery involvement in Moyamoya disease may result in infraction [41,42] which can cause problematic social adaption [42]. Simulation results showed that posterior cerebral artery involvement in Moyamoya disease is less in stage 4. Therefore, at that stage, a more favourable clinical and social outcome may be expected.

Coarctation of the aorta causes upper-limb hypertension [10,11]. The simulation results showed that aortic pressure increased in the adult and child cardiovascular models simulating coarctation of the aorta. The cerebral blood flow rate increases in patients with coarctation of the aorta [43]. The total cerebral blood flow increased to 926 mL/min and 1421 mL/min in the adult and child cardiovascular system models simulating coarctation of the aorta due to increased aortic pressure. The increased cerebral blood flow rate may increase the risk of developing an aneurysm and bleeding even in the early stages of Moyamoya disease due to increased pressure. Furthermore, an increase in systolic and diastolic blood pressure may increase the risk of ischemic stroke in the early stages of Moyamoya disease for adults and children. The percentage increase in systolic blood pressure was more noticeable than that in diastolic blood pressure in adult and child cardiovascular system models. Thus, the risk of ischemic stroke may be higher in Moyamoya disease with coarctation of the aorta when occlusion occurs in the posterior cerebral circulation.

Moyamoya disease with coarctation of the aorta is generally reported in case reports as in [9,11,44,45]. Altered blood flow rates in cerebral arteries due to coarctation of the aorta can be simulated in patients of different ages by modifying the parameter values accordingly. Again, the outputs of the presented model can be used as personalised boundary conditions in CFD models for patients with Moyamoya disease and coarctation of the aorta. Simulation of coarctation of the aorta shows the capabilities of the presented numerical model.

Volume curves over diastolic phase in the left atrium and ventricle appear to be linear due to the utilised equation describing the left-ventricular pressure, linear left-atrial model pressure–volume relation, and heart valve models. This can be considered as a limitation of the study.

## 5. Conclusions

In this study, a numerical model was utilised to simulate the cerebral blood flow rate in adults and children aged between 8 and 12 for a healthy condition and Moyamoya disease with and without coarctation of the aorta. The presented numerical model in this study can simulate blood flow rates in cerebral circulation and provide insights into cerebral perfusion at different stages of Moyamoya disease. The numerical model utilised in this study can simulate different stages of Moyamoya disease and evaluate the effects of the advancing stages of Moyamoya disease in adults and children.

## Figures and Tables

**Table 1:** Systolic and diastolic aortic blood pressures (SBP and DBP), cardiac output (CO), total cerebral blood flow rate (CBF), and mean cerebral blood flow rates in the internal carotid, vertebral, basilar, anterior, middle, and posterior arteries (ICA, VA, BA, ACA, MCA, and PCA) in the adult and child cardiovascular system models simulating healthy conditions and advancing stages of the Moyamoya disease with and without coarctation of the aorta.

	Healthy		Stage 1		Stage 2		Stage 3		Stage 4	
	Adult	Child	Adult	Child	Adult	Child	Adult	Child	Adult	Child
SBP [mmHg]	119	102	119	103	120	105	121	106	123	110
DBP [mmHg]	78	58	79	58	79	60	80	61	82	65
CO [L/min]	5.06	4.60	5.05	4.59	5.04	4.57	5.03	4.56	5.01	4.50
CBF [mL/min]	724	1072	676	1007	627	892	571	831	444	537
ICA [mL/min]	278	412	222	330	200	263	154	212	49	58
VA [mL/min]	84	124	117	174	113	183	131	203	173	210
BA [mL/min]	168	249	233	347	227	366	263	406	346	420
ACA [mL/min]	91	134	72	108	95	118	12	16	0.77	2.69
MCA [mL/min]	141	209	113	168	71	102	94	137	53	54
PCA [mL/min]	50	73	70	104	89	144	116	169	104	103
SBP_Co_ [mmHg]	147	133	148	135	150	138	151	139	154	147
DBP_Co_ [mmHg]	84	64	84	66	85	68	86	69	89	76
CO_Co_ [L/min]	4.79	4.58	4.78	4.56	4.76	4.53	4.75	4.52	4.71	4.44
CBF_Co_ [mL/min]	926	1421	867	1341	806	1197	735	1121	576	741
ICA_Co_ [mL/min]	356	546	284	439	257	353	198	286	63	81
VA_Co_ [mL/min]	107	165	149	231	146	246	169	274	225	290
BA_Co_ [mL/min]	215	330	299	462	291	491	339	548	449	579
ACA_Co_ [mL/min]	116	178	93	144	121	158	16	21	0.99	3.71
MCA_Co_ [mL/min]	181	278	145	224	91	137	122	185	69	74
PCA_Co_ [mL/min]	63	97	89	138	115	194	149	228	134	142

## Data Availability

Not applicable.

## Supplementary Materials

The following supporting information can be downloaded at https://www.mdpi.com/article/10.3390/bioengineering10010077/s1: Table S1. The parameters of left and right atria and ventricles in the adult and child cardiovascular system models; Table S2. The parameters used in the systemic and pulmonary circulatory system; Table S3. The circle of Willis parameters used in the adult cardiovascular system model; Table S4: The circle of Willis parameters used in the child cardiovascular system model.
