# Supplementary material for: Computational Modelling of Cerebral Blood Flow Rate at Different Stages of Moyamoya Disease in Adults and Children
Source: Bioengineering (Basel). 2023 Jan 6;10(1):77. doi: 10.3390/bioengineering10010077 (PMC9854682; doi:10.3390/bioengineering10010077)
Supplement: Supplementary file 1 [file bioengineering-10-00077-s001.zip › bioengineering-2062710-supplementary.pdf]

Table S1: The parameters of left and right atriums and ventricles in the adult and child cardiovascular system models.

	Left Ventricle		Right Ventricle		Left Atrium		Right Atrium	
	**Adult**	**Child**	**Adult**	**Child**	**Adult**	**Child**	**Adult**	**Child**
A	1	1	1	1	-	-	-	-
B	0.0252	0.024	0.0252	0.024	-	-	-	-
D [s]	-	-	-	-	0.05×T	0.05×T	0.05×T	0.05×T
E_{max} [mmHg/mL]	2.5	3.5	1	1.4	0.3	0.4	0.3	0.4
E_{min} [mmHg/mL]	-	-	-	-	0.2	0.2	0.2	0.2
K	1.15	1.5	1.75	3.25	1.20	2.5	1.20	2.5
l [cm]	8	7	8	7	5.5	4.5	5.5	4.5
T [s]	0.8	0.75	0.8	0.75	0.8	0.75	0.8	0.75
T_a [s]	-	-	-	-	0.8×T	0.8×T	0.8×T	0.8×T
T_1 [s]	0.33×T	0.33×T	0.33×T	0.33×T	-	-	-	-
T_2 [s]	0.45×T	0.45×T	0.45×T	0.45×T	-	-	-	-
V_0 [mL]	15	10	40	25	5	5	5	5

Table S2: The parameters used in the systemic and pulmonary circulatory system. R, L and C denote resistance, inertance and compliance of blood vessels (The values in the brackets denote the parameter values in the simulation of coarctation of the aorta).

	R [mmHg s/mL]		C [mL/mmHg]		L [mmHg s^2/mL]	
	**Adult**	**Child**	**Adult**	**Child**	**Adult**	**Child**
Aorta	0.01	0.02	0.04 (0.02)	0.10 (0.008)	1e-4	1e-5
Aortic Arch	0.05	0.05	0.25 (0.12)	0.20 (0.08)	1e-4	1e-5
Systemic Arterioles	0.75 (1.20)	0.75 (1.25)	2 (0.7)	1.1 (0.6)	1e-4	1e-5
Systemic Capillaries	0.24	0.24	4	2	-	-
Systemic Veins	0.10	0.12	30	17	-	-
Pulmonary Arteries	0.02	0.02	3	2.2	1e-4	1e-5
Pulmonary Arterioles	0.10	0.10	6	3.4	1e-4	1e-5
Pulmonary Veins	0.10	0.12	30	17	-	-
Mitral Valve	0.0025	0.0025	-	-	-	-
Aortic Valve	0.0025	0.0025	-	-	-	-
Tricuspid Valve	0.001	0.001	-	-	-	-
Pulmonary Valve	0.001	0.001	-	-	-	-

Table S3: The circle of Willis parameters used in the adult cardiovascular system model. R, L and C denote resistance, inertance and compliance of blood vessels, S represents stage. (**ICA:** Internal Carotid Arteries, **VA:** Vertebral Arteries, **BA:** Basilar Artery, **ACA:** Anterior Cerebral Arteries, **ACHA:** Anterior Choroidal Arteries, **ACOA:** Anterior Communicating Artery, **MCA:** Middle Cerebral Arteries, **PCA:** Posterior Cerebral Arteries, **PCOA:** Posterior Communicating Arteries, **SCA:** Superior Cerebellar Arteries, **OA:** Ophthalmic Arteries, **PC:** Pial Arterioles, **CC:** Cerebral Capillaries, **VC:** Cerebral Veins).

	**R [mmHg s/mL]**					**C [mL/mmHg]**	**L [mmHg s^2/mL]**
	**S0**	**S1**	**S2**	**S3**	**S4**		
ICA	1.74	4.34	6.26	7.82	60.82	-	1e-4
VA	5.00	5.00	5.00	5.00	5.00	-	1e-4
BA	6.47	4.53	6.47	6.47	0.65	1e-3	-
ACA1	9.76	9.76	5.85	97.61	117.13	-	-
ACA2	4.18	4.18	4.18	4.18	6.27	1e-3	-
ACHA	38.89	38.89	38.89	38.89	38.89	-	-
ACOA	53.57	53.57	53.73	53.57	53.57	-	-
MCA	8.94	8.94	13.41	13.41	1.79	1e-3	-
PCA1	0.82	0.82	0.33	0.16	8.21	1e-3	-
PCA2	3.88	3.88	1.55	0.77	11.63	1e-3	-
PCOA	321.43	321.43	257.14	257.14	128.57	-	-
SCA	7.14	7.14	7.14	7.14	28.57	-	-
OA	125.00	125.00	125.00	125.00	125.00	1e-3	-
R	0.018	0.018	0.018	0.018	0.018	-	-
PC	3.60	3.60	3.60	3.60	3.60	0.50	-
CC	0.10	0.10	0.10	0.10	0.10	2.00	-
VC	0.10	0.10	0.10	0.10	0.10	6.00	-

Table S4: The circle of Willis parameters used in the child cardiovascular system model. R, L and C denote resistance, inertance and compliance of blood vessels, S represents stage. (**ICA:** Internal Carotid Arteries, **VA:** Vertebral Arteries, **BA:** Basilar Artery, **ACA:** Anterior Cerebral Arteries, **ACHA:** Anterior Choroidal Arteries, **ACOA:** Anterior Communicating Artery, **MCA:** Middle Cerebral Arteries, **PCA:** Posterior Cerebral Arteries, **PCOA:** Posterior Communicating Arteries, **SCA:** Superior Cerebellar Arteries, **OA:** Ophthalmic Arteries, **PC:** Pial Arterioles, **CC:** Cerebral Capillaries, **VC:** Cerebral Veins).

	**R [mmHg s/mL]**					**C [mL/mmHg]**	**L [mmHg s^2/mL]**
	**S0**	**S1**	**S2**	**S3**	**S4**		
ICA	0.96	2.39	4.97	5.93	47.78	-	1e-5
VA	2.75	2.75	2.75	2.75	2.75	-	1e-5
BA	3.56	2.49	3.56	3.56	1.78	6e-4	-
ACA1	5.37	5.37	3.22	53.68	75.16	-	-
ACA2	2.30	2.30	2.30	2.30	3.45	6e-4	-
ACHA	21.39	21.39	21.39	21.39	21.39	-	-
ACOA	29.46	29.46	29.46	29.46	29.46	-	-
MCA	4.92	4.92	6.39	6.39	3.93	6e-4	-
PCA1	0.45	0.45	0.18	0.14	6.78	6e-4	-
PCA2	2.13	2.13	0.85	0.63	10.66	6e-4	-
PCOA	176.79	176.79	141.43	141.43	70.71	-	-
SCA	3.93	3.93	3.93	3.93	3.93	-	-
OA	68.75	68.75	68.75	68.75	68.75	6e-4	-
R	0.0098	0.0098	0.0098	0.0098	0.0098	-	-
PC	1.98	1.98	1.98	1.98	1.98	0.10	-
CC	0.055	0.055	0.055	0.055	0.055	0.70	-
VC	0.055	0.055	0.055	0.055	0.055	2.20	-
